# Association Between Mandibular Ramus Morphometry and Mandibular Third Molar Impaction: A Systematic Review and Meta‐Analysis

**DOI:** 10.1155/ijod/4403436

**Published:** 2026-08-27

**Authors:** Nazanin Salmani, Hooman Shafaee, Mostafa Abtahi, Shayan Yousefi, Amirhossein Rahat Dahmardeh, Seyed MohammadMobin Mousavi, Mahdi Naseri Takaloo, Fatemeh Elahi

**Affiliations:** ^1^ Student Research Committee, Faculty of Dentistry, Mashhad University of Medical Sciences, Mashhad, Iran, mums.ac.ir; ^2^ Patient Safety Research Center, Clinical Research Institute, Mashhad University of Medical Sciences, Mashhad, Iran, mums.ac.ir; ^3^ Department of Orthodontic, Dental Research Center, School of Dentistry, Mashhad University of Medical Sciences, Mashhad, Iran, mums.ac.ir; ^4^ Faculty of Medicine, Mashhad University of Medical Sciences, Mashhad, Iran, mums.ac.ir; ^5^ Faculty of Dentistry, Tehran University of Medical Sciences, Tehran, Iran, tums.ac.ir

**Keywords:** mandibular ramus, mandibular third molar, meta-analysis, panoramic radiography, ramus height, ramus width, systematic review, third molar impaction

## Abstract

**Background:**

Mandibular third molar impaction is a common clinical condition associated with various functional and pathological complications. Skeletal and morphometric characteristics of the mandible, particularly mandibular ramus dimensions, have been proposed as potential contributors to impaction risk; however, existing evidence remains inconsistent.

**Objective:**

To systematically review and quantitatively synthesize the available evidence on the association between mandibular ramus dimensions (height and width) and mandibular third molar impaction.

**Methods:**

A systematic review and meta‐analysis were conducted in accordance with Preferred Reporting Items for Systematic Reviews and Meta‐Analyses (PRISMA) guidelines, with the protocol prospectively registered in PROSPERO (Registration ID: CRD420251238995). PubMed, Embase, Web of Science, Scopus, and Google Scholar were searched from inception to October 16, 2025. Observational studies evaluating the relationship between mandibular ramus height and/or width and mandibular third molar impaction were included. Meta‐analyses were performed using random‐effects models with restricted maximum‐likelihood (REML) estimation and Hartung–Knapp adjustment. Ramus height and width were analyzed separately as mean differences (MDs) with 95% confidence intervals (CIs). Subgroup analyses by study design and sensitivity analyses were conducted to explore heterogeneity. Risk of bias was assessed using the Joanna Briggs Institute (JBI) Critical Appraisal Checklist.

**Results:**

Eleven studies comprising 3570 participants were included, of which nine contributed to the meta‐analysis of ramus height and six to ramus width. Meta‐analysis demonstrated a significantly smaller mandibular ramus height in individuals with impacted mandibular third molars compared with erupted controls (MD = −1.03 mm; 95% CI: −1.85 to −0.21; *p* = 0.020), with substantial heterogeneity (*I* ^2^ = 98.0%). In contrast, no statistically significant difference was observed for ramus width (MD = 0.66 mm; 95% CI: −0.74 to 2.06; *p* = 0.281). Subgroup analyses by study design did not demonstrate significant subgroup differences for ramus height but indicated significant between‐subgroup variability for ramus width. Sensitivity analyses generally supported the robustness of the findings, although statistical significance for ramus height was attenuated after omission of several influential studies. Eight studies were judged to have low risk of bias and three moderate risk.

**Conclusion:**

Reduced mandibular ramus height may be more consistently associated with mandibular third molar impaction than ramus width, although substantial heterogeneity limits definitive conclusions. While sexual dimorphism in ramus morphology is evident, its influence on impaction risk remains unclear. These findings highlight the need for standardized measurement protocols and well‐designed longitudinal studies to further clarify the role of mandibular ramus morphology in third molar impaction.

## 1. Introduction

Third molar impaction is a common clinical finding worldwide, with reported prevalence varying considerably across populations and demographic groups, ranging from ~3.8% to 70% [1–3]. Mandibular third molars are more frequently impacted than maxillary third molars [4, 5]. Impacted third molars are associated with a range of functional and pathological complications, including pericoronitis, caries of adjacent teeth, external root resorption, cystic degeneration, and increased surgical complexity. These potential consequences underscore the clinical importance of early identification and risk assessment of third molar impaction [6–10].

The etiology of mandibular third molar impaction is multifactorial. Common contributing factors include abnormal orientation of the developing tooth germ, insufficient space within the dental arch, the presence of supernumerary teeth, ankylosis of primary or permanent teeth, and inadequate bone resorption related to local or systemic conditions [11]. Among these factors, skeletal and morphometric characteristics of the mandible have received increasing attention as potential contributors to impaction risk.

Several radiographic studies have investigated whether mandibular ramus morphology influences the eruption or impaction of mandibular third molars, with particular emphasis on ramus height and ramus width [12–15]. Multiple authors have reported significantly greater ramus height in individuals with erupted third molars compared with those with impacted teeth [14, 16, 17], whereas other studies have observed no significant association between ramus height and impaction status [18, 19]. Findings related to ramus width have been similarly inconsistent, with some studies reporting greater width in erupted cases [14, 19] and others identifying greater width in impacted groups [17, 20]. Only a limited number of studies have examined ramus dimensions in relation to impaction classifications, such as Pell and Gregory or Winter’s systems [21, 22]. Furthermore, sex‐specific analyses of ramus morphology and impaction remain insufficiently explored.

Despite the growing body of literature, interpretation of existing findings is complicated by substantial methodological heterogeneity, including variation in measurement landmarks, imaging modalities, study designs, and limited adjustment for confounding variables. These limitations have contributed to inconsistent and sometimes conflicting results across studies, highlighting the need for a rigorous synthesis of available evidence.

Accordingly, the aim of the present systematic review and meta‐analysis was to quantitatively evaluate the association between mandibular ramus dimensions and mandibular third molar impaction. Based on prevailing trends in the literature, it was hypothesized that impacted mandibular third molars would be associated with reduced ramus height and potentially reduced ramus width.

## 2. Methods and Materials

This systematic review was conducted and reported in accordance with the Preferred Reporting Items for Systematic Reviews and Meta‐Analyses (PRISMA) guidelines. The protocol was prospectively registered in the PROSPERO database (Registration ID: CRD420251238995).

### 2.1. Eligibility Criteria

This review aimed to identify, appraise, and synthesize observational studies evaluating the association between mandibular ramus morphometric dimensions and mandibular third molar impaction.

#### 2.1.1. Inclusion Criteria

Studies were eligible if they met the following criteria:

• Population: Human participants undergoing radiographic assessment (panoramic radiographs, lateral cephalograms, or other imaging modalities) of mandibular third molars, including both impacted and erupted teeth.
• Exposure: Assessment of at least one mandibular ramus morphometric variable (ramus height or ramus width).
• Comparator:
  ° Impacted versus erupted mandibular third molars;
  ° Different impaction types (e.g., mesioangular, vertical, horizontal, and distoangular); and/or
  ° Correlation analyses between ramus dimensions and impaction characteristics (e.g., severity, angulation, or position).
• Outcome: Quantitative measures describing the association between ramus dimensions and third molar impaction, including mean differences (MDs), correlation coefficients, odds ratios, or regression parameters.
• Study design: Observational studies.

#### 2.1.2. Exclusion Criteria

Studies were excluded if they:

• Focused solely on angular parameters (e.g., gonial angle and mandibular plane angle).
• Reported skeletal classifications only (Class I, II, or III) without ramus morphometry.
• Lacked quantitative measurements of ramus dimensions.
• Included only maxillary third molars.
• Were conducted on animals, cadavers, syndromic or craniofacial anomaly cases, or edentulous mandibles.
• Lacked a comparator or correlation analysis, or merely described average ramus measurements without linking them to impaction or eruption status.

The primary outcome was the statistical association between mandibular ramus height and/or width and mandibular third molar impaction.

### 2.2. Search Strategy

A comprehensive literature search was conducted in PubMed, Embase (via Elsevier), Web of Science, Scopus, and Google Scholar from database inception to October 16, 2025. Search strategies combined controlled vocabulary terms (MeSH/Emtree) and free‐text keywords related to “third molar,” “impaction,” and “mandibular ramus.” Detailed search strategies for each database are provided in Table 1.

**Table 1:** Search strategies used for databases.

Search terms
**PubMed: 704 results** (“molar, third"[MeSH Terms] OR “third molar"[Title/Abstract] OR “wisdom tooth"[Title/Abstract] OR “mandibular third molar"[Title/Abstract] OR “wisdom teeth"[Title/Abstract] OR “third molars"[Title/Abstract] OR “lower third molar"[Title/Abstract] OR “lower wisdom tooth"[Title/Abstract] OR “M3"[Title/Abstract]) AND (“tooth, impacted"[MeSH Terms] OR “impaction"[Title/Abstract] OR “impacted"[Title/Abstract] OR “impacted tooth"[Title/Abstract] OR “impacted teeth"[Title/Abstract] OR “Pell and Gregory"[Title/Abstract] OR “Winter"[Title/Abstract]) AND (“gonial angle"[Title/Abstract] OR “mandibular plane"[Title/Abstract] OR “mandibular plane angle"[Title/Abstract] OR “ramus"[Title/Abstract] OR “ramus width"[Title/Abstract] OR “ramus height"[Title/Abstract] OR “mandibular ramus"[Title/Abstract] OR “ramus depth"[Title/Abstract] OR “ramus length"[Title/Abstract] OR “cephalometric"[Title/Abstract] OR “cephalometry"[Title/Abstract] OR “lateral cephalometry"[Title/Abstract] OR “panoramic"[Title/Abstract] OR “OPG"[Title/Abstract] OR “orthopantomography"[Title/Abstract] OR “orthopantomogram"[Title/Abstract]) **Embase: 185 results** (’third molar’/exp OR ’mandibular third molar’/exp OR ’wisdom tooth’/exp OR ’third molar’:ti,ab,kw OR ’mandibular third molar’:ti,ab,kw OR ’lower third molar’:ti,ab,kw OR ’wisdom tooth’:ti,ab,kw OR m3:ti,ab,kw) AND (impact ^∗^:ti,ab,kw OR impacted:ti,ab,kw OR impaction:ti,ab,kw OR unerupted:ti,ab,kw OR ’tooth impaction’/exp OR ’tooth impaction’:ti,ab,kw) AND (’gonial angle’:ti,ab,kw OR gonial:ti,ab,kw OR ’mandibular plane’:ti,ab,kw OR ’mandibular plane angle’:ti,ab,kw OR ’mandibular plane inclination’:ti,ab,kw OR ’ramus’:ti,ab,kw OR ’ramus width’:ti,ab,kw OR ’ramus height’:ti,ab,kw OR ’ramus length’:ti,ab,kw OR ’ramus depth’:ti,ab,kw OR ’ramus breadth’:ti,ab,kw OR ’mandibular ramus’:ti,ab,kw OR ’ramus measurement’:ti,ab,kw) **Web of Science: 164 results** (((((((ALL = (“third molar”)) OR ALL = (“wisdom tooth”)) OR ALL = (“mandibular third molar”)) OR ALL = (“wisdom teeth”)) OR ALL = (“third molars”)) OR ALL = (“lower third molar”)) OR ALL = (“lower wisdom tooth”)) OR ALL = (M3) AND (((((ALL = (“impaction”)) OR ALL = (“impacted”)) OR ALL = (“impacted tooth”)) OR ALL = (“impacted teeth”)) OR ALL = (“Pell and Gregory”)) OR ALL = (“Winter”) AND ((((((((ALL = (“gonial angle”)) OR ALL = (“mandibular plane”)) OR ALL = (“mandibular plane angle”)) OR ALL = (“ramus”)) OR ALL = (“ramus width”)) OR ALL = (“ramus height”)) OR ALL = (“mandibular ramus”)) OR ALL = (“ramus depth”)) OR ALL = (“ramus length”) **Scopus: 236 results** (TITLE‐ABS‐KEY (“third molar”) OR TITLE‐ABS‐KEY (“wisdom tooth”) OR TITLE‐ABS‐KEY (“mandibular third molar”) OR TITLE‐ABS‐KEY (“wisdom teeth”) OR TITLE‐ABS‐KEY (“third molars”) OR TITLE‐ABS‐KEY (“lower third molar”) OR TITLE‐ABS‐KEY (“lower wisdom tooth”) OR TITLE‐ABS‐KEY (“M3”)) AND (TITLE‐ABS‐KEY (“impaction”) OR TITLE‐ABS‐KEY (“impacted”) OR TITLE‐ABS‐KEY (“impacted tooth”) OR TITLE‐ABS‐KEY (“impacted teeth”) OR TITLE‐ABS‐KEY (“Pell and Gregory”) OR TITLE‐ABS‐KEY (“Winter”)) AND (TITLE‐ABS‐KEY (“gonial angle”) OR TITLE‐ABS‐KEY (“mandibular plane”) OR TITLE‐ABS‐KEY (“mandibular plane angle”) OR TITLE‐ABS‐KEY (“ramus”) OR TITLE‐ABS‐KEY (“ramus width”) OR TITLE‐ABS‐KEY (“ramus height”) OR TITLE‐ABS‐KEY (“mandibular ramus”) OR TITLE‐ABS‐KEY (“ramus depth”) OR TITLE‐ABS‐KEY (“ramus length”)) **Google Scholar: 170 results** allintitle: (“mandibular third molar” OR “lower third molar”) impaction AND (“gonial angle” OR “mandibular plane” OR “ramus width” OR “ramus height” OR “mandibular ramus”) ‐related articles were searched.

No restrictions were applied regarding language or publication year. The search was designed and executed by one author. To maximize study identification, two additional authors independently performed supplementary hand‐searching of reference lists and related records. Duplicate citations were removed prior to screening, and the reference lists and related records of included studies were examined manually to identify potentially eligible studies.

### 2.3. Study Selection

**Figure 1:**
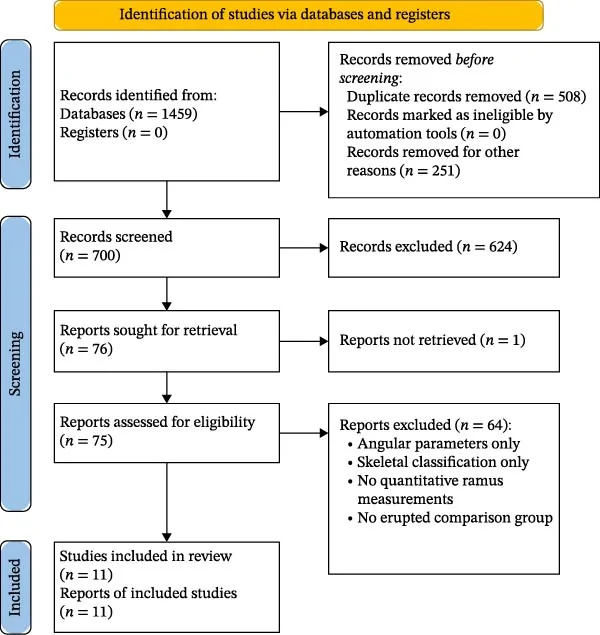
PRISMA flow diagram illustrating the study selection process.

Two reviewers independently evaluated the titles and abstracts of all retrieved records for potential eligibility. Full‐text articles considered potentially relevant were subsequently reviewed against the predefined eligibility criteria. Reference lists of included studies were also examined to identify additional eligible publications. Any disagreements were resolved through discussion until consensus was achieved. The study selection process is summarized in the PRISMA flow diagram (Figure 1).

Records categorized as “removed for other reasons” corresponded to studies that were excluded after deduplication during initial relevance assessment because they were clearly identified as nonoriginal research articles, including reviews, case reports, case series, conference abstracts, book chapters, and animal studies, and therefore did not proceed to full title and abstract screening.

### 2.4. Data Extraction

Data were independently extracted by two reviewers using a standardized form developed a priori and piloted on three included studies. Discrepancies were resolved through discussion and consensus. Extracted data included the following:

• Study characteristics: authors, publication year, country, and study design.
• Participants: number, mean age, and sex distribution.
• Exposure and comparators: type and measurement of ramus morphometric variables and comparator groups.
• Outcomes: effect estimates describing associations between ramus dimensions (height and width) and third molar impaction (e.g., correlation coefficients, MDs, odds ratios, and regression parameters).

### 2.5. Statistical Analysis

Quantitative synthesis was undertaken when at least two studies reported sufficiently comparable outcome measures. Mandibular ramus height and ramus width were analyzed separately. Continuous outcomes were pooled as MDs with 95% confidence intervals (CIs). Studies with incomplete or nonextractable variance measures were excluded from quantitative synthesis but were included in the qualitative synthesis when applicable.

When studies reported subgroup data (e.g., sex‐specific groups, eruption stages, or Pell and Gregory classifications), subgroups were combined into binary impacted and erupted categories to avoid unit‐of‐analysis errors and permit quantitative synthesis. Studies reporting multiple impacted categories (e.g., Pell and Gregory positions B and C or semierupted and impacted teeth) had these categories merged into a single impacted group. Combined means and standard deviations were calculated using standard formulas for weighted pooled means and pooled variances based on subgroup sample sizes, means, and standard deviations to obtain summary estimates for the pooled impacted and erupted groups.

In one study reporting fully erupted, semierupted, impacted, and partially developed teeth, fully erupted teeth were retained as the erupted group, whereas semierupted and impacted teeth were combined into a single impacted category. Teeth classified as partially developed were excluded from quantitative synthesis because they represent a developmental stage rather than an eruption or impaction outcome.

Meta‐analyses were conducted using the inverse‐variance method under a random‐effects model to account for anticipated clinical and methodological heterogeneity. Between‐study variance was estimated using the restricted maximum‐likelihood (REML) method, with Hartung–Knapp adjustment applied to calculate CIs. Statistical heterogeneity was assessed using the *I* ^2^ statistic, *τ* ^2^, and Cochran’s Q test, with *I* ^2^ values of ~25%, 50%, and 75% interpreted as low, moderate, and high heterogeneity, respectively. Prediction intervals were calculated to estimate the range within which the true effect may be expected to lie in future populations.

Given the limited number of prospective studies, observational studies of different designs (prospective, retrospective, case–control, and cross‐sectional) were included. Prespecified subgroup analyses were conducted according to study design to explore potential sources of heterogeneity. Sensitivity analyses were conducted using a leave‐one‐out approach to evaluate the influence of individual studies on the pooled estimates. Correlation findings were synthesized narratively because substantial variation existed in outcome definitions and in the anatomical measures used across studies.

Assessment of publication bias was not performed because fewer than 10 studies were included in each meta‐analysis, rendering funnel plots and statistical tests for funnel plot asymmetry potentially unreliable and underpowered. A two‐sided *p*‐value  < 0.05 was considered statistically significant. All analyses were performed using R (meta package).

### 2.6. Risk of Bias Assessment

Methodological quality was assessed using the Joanna Briggs Institute (JBI) Critical Appraisal Checklist for analytical cross‐sectional studies. Although the included studies were variably described as retrospective, prospective, cross‐sectional, or retrospective case–control investigations, they predominantly comprised radiographic observational studies evaluating mandibular ramus morphometry and mandibular third molar impaction status at a single assessment point. Therefore, the JBI analytical cross‐sectional checklist was selected to provide a consistent appraisal of participant selection, measurement validity, confounding factors, and statistical analyses across studies with comparable methodological characteristics. Two reviewers independently evaluated each study, with disagreements resolved by discussion. Studies were categorized as low (≥75%), moderate (50%–74%), or high (<50%) risk of bias based on the proportion of affirmative responses.

## 3. Results

This systematic review was conducted in accordance with the PRISMA guidelines and prospectively registered with PROSPERO (Registration ID: CRD420251238995).

### 3.1. Literature Search and Study Selection

Following a systematic search of PubMed, Scopus, Web of Science, Embase, and Google Scholar, a total of 1459 records were identified. After removal of duplicates, 700 records were screened based on titles and abstracts by two independent reviewers. Seventy‐six studies were retrieved for full‐text assessment, of which one full text could not be obtained. Full‐text eligibility screening was conducted independently by the same reviewers, with discrepancies resolved through consensus. Ultimately, 11 studies met the inclusion criteria and were included in this review [12–22]. The study selection process is illustrated in the PRISMA 2020 flow diagram (Figure 1).

### 3.2. Study Characteristics

Of the included studies, eight were published between 2020 and 2025 [12, 13, 15, 17–19, 21, 22] and three before 2020 [14, 16, 20]. The studies originated from India, Turkey, Iran, Saudi Arabia, China, Italy, and Singapore. Most employed retrospective case–control designs, with fewer prospective and cross‐sectional studies. All studies utilized panoramic radiographs, and one additionally incorporated lateral cephalometric imaging. Sample sizes ranged from 81 to 1125 participants, and all participants were older than 17 years, ensuring complete development of the mandibular third molar. Detailed study characteristics and methodologies are summarized in Supporting Information 1: Table S1.

### 3.3. Participant Characteristics

Across the included studies, a total of 3570 participants were evaluated, comprising ~48.3% males and 51.7% females. This number represents the sum of participants reported across the included studies and does not necessarily reflect a cohort of unique individuals as data were extracted at the study level. One study was identified as potentially overlapping with another publication from the same research group; however, only one of these reports was included in the quantitative synthesis to avoid duplication in the meta‐analysis. The proportion of impacted mandibular third molars ranged from 18.48% to 75.0%, while erupted third molars ranged from 25.0% to 81.52%.

Three studies reported sex‐stratified outcomes [15, 16, 20], with impaction rates ranging from 43.31% to 59.38% in males and 45.0% to 56.62% in females. Although inclusion and exclusion criteria varied slightly, most studies excluded individuals with syndromic conditions, craniofacial anomalies, surgically altered mandibles, a history of orthodontic or orthognathic treatment, trauma, cysts, or tumors.

### 3.4. Ramus Morphometric Variables

All included studies evaluated at least one mandibular ramus morphometric parameter, with eight studies assessing both ramus height and ramus width. Measurements were predominantly derived from orthopantomograms, with one study additionally using lateral cephalometric radiographs. Although landmark definitions varied slightly, ramus height was generally defined as the linear distance between the condylion and gonion, while ramus width was defined as the distance between the most convex points on the anterior and posterior borders of the ramus.

### 3.5. Associations Between Ramus Dimensions and Third Molar Impaction

Six studies reported a significant association between reduced ramus height and an increased likelihood of mandibular third molar impaction. One of these studies demonstrated significantly lower ramus height in both male and female participants with impacted third molars. In contrast, five studies reported no statistically significant difference in ramus height between impacted and erupted groups.

Regarding ramus width, three studies reported significantly lower values in impacted groups, while one study observed significantly reduced width in females but not in males. Three studies found no significant association between ramus width and third molar impaction, and one study reported a significantly greater ramus width in the impacted group.

Only one study examined impaction type using Winter’s classification (mesioangular, horizontal, and vertical positions) and reported no significant association between ramus dimensions and impaction type.

### 3.6. Meta‐Analysis Results

#### 3.6.1. Meta‐Analysis of Ramus Height

Nine studies were included in the meta‐analysis of mandibular ramus height after exclusion of one potentially overlapping cohort publication [13, 14, 16–22]. One additional eligible study [12] was excluded from quantitative synthesis due to incomplete or nonextractable variance data required for pooling, although it was included in qualitative synthesis.

**Figure 2:**
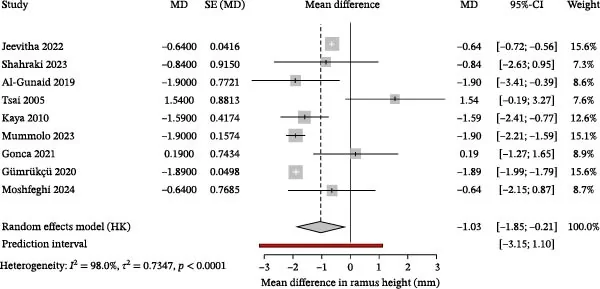
Forest plot of the meta‐analysis examining mandibular ramus height.

Random‐effects meta‐analysis using the REML estimator with Hartung–Knapp adjustment demonstrated a significantly smaller ramus height in individuals with impacted mandibular third molars compared with erupted controls (mean difference [MD] = −1.03 mm; 95% CI: −1.85 to −0.21; *p* = 0.020). Substantial heterogeneity was observed (*I* ^2^ = 98.0%; *τ* ^2^ = 0.73; *p*  < 0.0001). The prediction interval ranged from −3.15 to 1.10 mm, indicating considerable variability in the true effect across populations. The prediction interval provides an estimate of the range in which the true effect may lie in future studies and therefore reflects the extent of between‐study variability beyond the confidence interval. Figure 2 presents the forest plot of the meta‐analysis examining mandibular ramus height.

#### 3.6.2. Meta‐Analysis of Ramus Width

**Figure 3:**
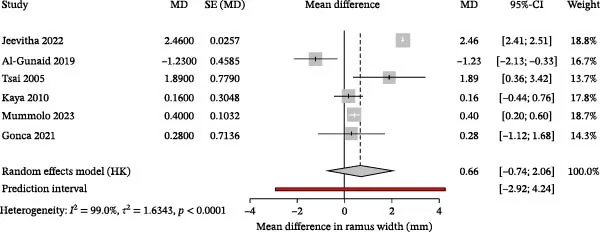
Forest plot of the meta‐analysis examining mandibular ramus width.

Six studies were included in the meta‐analysis of mandibular ramus width [14, 16–20]. The pooled random‐effects estimate showed no statistically significant difference between impacted and erupted groups (MD = 0.66 mm; 95% CI: −0.74 to 2.06; *p* = 0.281). Heterogeneity was substantial (*I* ^2^ = 99.0%; *τ* ^2^ = 1.63; *p*  < 0.0001), with a wide prediction interval (−2.92 to 4.24 mm). Figure 3 presents the forest plot of the meta‐analysis examining mandibular ramus width.

#### 3.6.3. Subgroup Analysis by Study Design

**Figure 4:**
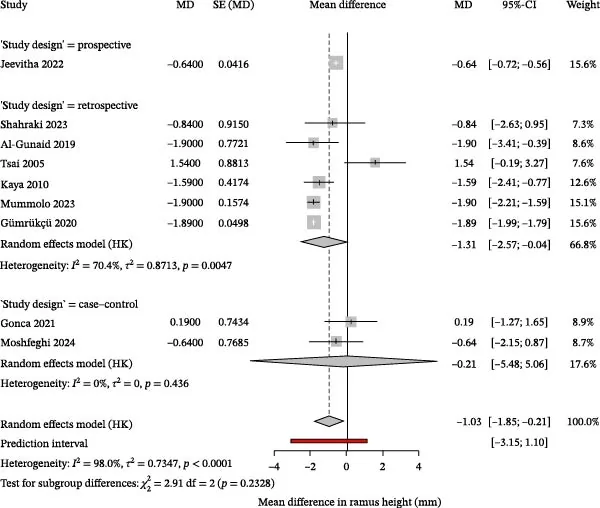
Subgroup analysis by study design for mandibular ramus height.

Subgroup analysis by study design did not demonstrate statistically significant between‐subgroup differences for mandibular ramus height (*Q* = 2.91, df = 2, *p* = 0.2328). The retrospective subgroup (*k* = 6) demonstrated a statistically significant reduction in ramus height among individuals with impacted mandibular third molars (MD = −1.31 mm; 95% CI: −2.57 to −0.04), whereas the case–control subgroup did not show a significant association (MD = −0.21 mm; 95% CI: −5.48 to 5.06). The single prospective study also reported a negative mean difference (MD = −0.64 mm; 95% CI: −0.72 to −0.56), but this subgroup was insufficient for pooled inference. Despite subgrouping, substantial residual heterogeneity persisted within retrospective studies (*I* ^2^ = 70.4%). Subgroup analysis by study design for mandibular ramus height is shown in Figure 4.

**Figure 5:**
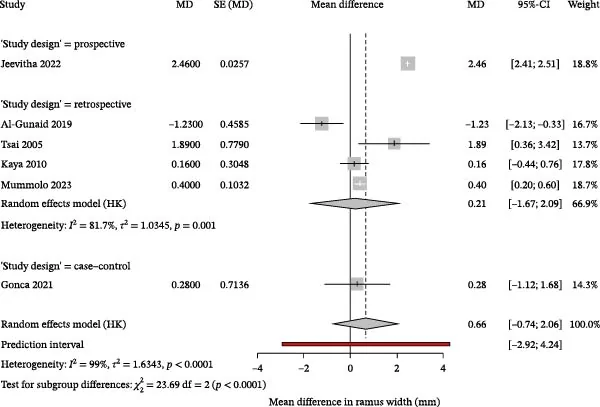
Subgroup analysis by study design for mandibular ramus width.

For mandibular ramus width, subgroup analysis demonstrated significant between‐group differences by study design (*Q* = 23.69, df = 2, *p*  < 0.0001). The retrospective subgroup did not demonstrate a statistically significant association (MD = 0.21 mm; 95% CI: −1.67 to 2.09), and the single prospective and case–control studies were insufficient for pooled subgroup inference. These findings suggest that methodological differences may contribute to heterogeneity but do not support a consistent relationship between ramus width and mandibular third molar impaction. Corresponding subgroup analyses for ramus width are provided in Figure 5.

#### 3.6.4. Sensitivity Analysis

Leave‐one‐out sensitivity analyses demonstrated that the pooled estimates were generally robust to sequential omission of individual studies. For mandibular ramus height, omission of most studies resulted in pooled estimates that remained statistically significant and consistently favored smaller ramus height among impacted third molars. However, exclusion of Kaya et al. [16], Mummolo et al. [17], or Gümrükçü et al. [21] yielded CIs that crossed the null value, although the direction and magnitude of the pooled effect remained largely unchanged. For mandibular ramus width, sequential omission of individual studies did not materially alter the nonsignificant pooled estimate, with all leave‐one‐out analyses yielding CIs encompassing the null effect. These findings suggest that the overall conclusions are reasonably robust despite substantial between‐study heterogeneity (Supporting Information 2: Figure S1 and Supporting Information 3: Figure S2).

### 3.7. Risk of Bias Assessment

**Figure 6:**
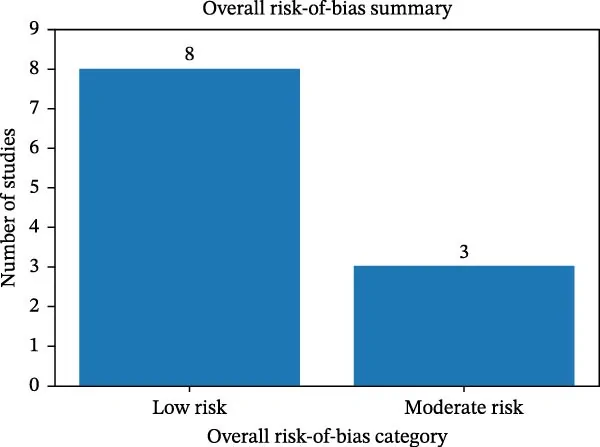
Summary of overall risk of bias assessment of included studies. Studies were categorized as low risk (≥75% affirmative responses), moderate risk (50%–74%), or high risk (<50%) according to the proportion of positive responses across the eight JBI domains.

**Figure 7:**
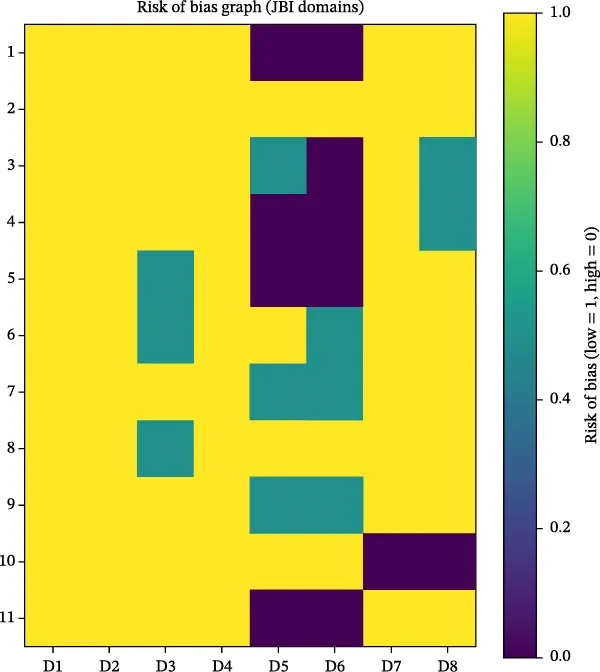
Domain‐level risk of bias graph of included studies. Domain abbreviations: D1, inclusion criteria clearly defined; D2, study subjects and setting described in detail; D3, exposure measured in a valid and reliable way; D4, outcomes measured in a valid and reliable way; D5, confounding factors identified; D6, strategies to address confounding factors stated; D7, outcomes measured objectively and consistently; D8, appropriate statistical analysis used.

Based on the JBI Critical Appraisal Checklist, eight studies were classified as having low risk of bias and three studies as having moderate risk. No studies were rated as high‐risk. Common methodological limitations included unclear measurement reliability, insufficient adjustment for confounding variables, and limited reporting of statistical correlation analyses. The risk of bias summary and graphical presentation are shown in Figures 6 and 7, while item‐level responses for each JBI domain are provided in Supporting Information 4: Table S2.

The JBI Critical Appraisal Checklist was applied across all included observational study designs, including retrospective case–control studies, to ensure consistency in risk‐of‐bias assessment. This approach is acknowledged as a methodological consideration given that different observational study designs may have distinct sources of bias.

## 4. Discussion

This systematic review synthesized evidence from 11 observational studies investigating the association between mandibular ramus morphometric dimensions and mandibular third molar impaction. Ramus height and ramus width were the most frequently evaluated anatomical parameters, although considerable variability existed in imaging modalities, landmark definitions, and analytical approaches. Despite this methodological diversity, several consistent patterns emerged when both qualitative synthesis and quantitative meta‐analysis were considered.

Ramus height demonstrated the most consistent association with mandibular third molar impaction. At the study level, six investigations reported significantly lower ramus height in individuals with impacted mandibular third molars. İbişoğlu and Uslu‐Akcam [12] observed significantly greater ramus height in erupted teeth compared with impacted ones, while similar findings were reported by Al‐Gunaid et al. [14] and Kaya et al. [16]. Mummolo et al. [17] and Jeevitha et al. [18] reported greater ramus height in erupted molars, although these differences did not reach statistical significance. In contrast, Moshfeghi et al. [22] and Gonca et al. [19] found no significant differences between impacted and erupted groups, while Shahraki et al. [13] observed significant differences only in partially developed molars. Collectively, these findings indicate that reduced ramus height frequently corresponds with third molar impaction, although the association is not uniformly observed across all populations and study designs.

Importantly, the meta‐analysis provided quantitative confirmation of this pattern. Pooled analysis demonstrated a statistically significant reduction in ramus height among individuals with impacted mandibular third molars despite substantial heterogeneity across studies. This finding supports the hypothesis that reduced vertical ramus dimensions may contribute to spatial limitations affecting third molar eruption, while also emphasizing that ramus height represents one component of a multifactorial anatomical framework rather than a deterministic predictor.

Although the pooled mean difference in ramus height was statistically significant, its magnitude was relatively small (1.03 mm), and its clinical significance should be interpreted with caution. Given the absence of established threshold values linking ramus height differences to impaction risk and the substantial heterogeneity observed among studies, this finding is unlikely to serve as an independent clinical predictor, particularly in view of the inherent limitations of panoramic radiography, including image magnification and potential landmark identification error. Nevertheless, reduced ramus height may represent an adjunctive anatomical indicator that could aid radiographic assessment and risk stratification when considered alongside other anatomical and developmental factors influencing mandibular third molar eruption.

Furthermore, the prediction interval for ramus height crossed the null value, indicating that future studies may observe a range of effects, including no association between ramus height and mandibular third molar impaction. This finding underscores the substantial between‐study variability and suggests that future studies conducted in different populations and methodological settings may yield a range of findings, including no association between ramus height and mandibular third molar impaction.

In contrast, the findings related to ramus width were markedly heterogeneous. Several studies reported significantly greater ramus width in erupted groups [14, 16, 19], while Jeevitha et al. [18] reported greater width in erupted teeth despite an absence of a significant correlation. Conversely, Tsai [20] reported significantly greater ramus width in impacted cases, and Mummolo et al. [17] observed slightly greater width among impacted teeth without statistical significance. Other studies reported no meaningful association between ramus width and impaction status [12, 18]. These inconsistent results were reflected in the meta‐analysis, which did not demonstrate a statistically significant pooled difference in ramus width between impacted and erupted groups. Together, these findings suggest that ramus width is a less reliable and less consistent morphological indicator of mandibular third molar impaction compared with ramus height. Similarly, the prediction interval for ramus width encompassed both negative and positive values, further supporting the lack of a consistent relationship between ramus width and mandibular third molar impaction across populations.

Sex‐stratified analyses further highlighted the presence of sexual dimorphism in mandibular ramus dimensions, although associations with impaction status remained inconsistent. Al‐Gunaid [15] reported significantly greater ramus height in erupted compared with impacted molars among men, while women demonstrated significantly greater values for both height and width in erupted teeth. In contrast, Tsai [20] found significantly greater ramus width in impacted male and female participants, with no significant association for height. Kaya et al. [16] reported generally larger ramus dimensions in men compared with women across both erupted and impacted groups. These findings confirm the presence of sexual dimorphism in ramus morphology; however, the influence of sex on third molar impaction risk appears to be variable and context‐dependent, likely interacting with other anatomical and developmental factors.

Only a limited number of studies evaluated ramus dimensions across impaction classifications. Gümrükçü et al. [21] reported significantly greater ramus height in Pell and Gregory Class A compared with Classes B and C, suggesting that more superficially positioned third molars may be associated with greater vertical ramus dimensions. However, the same study found no significant differences using Winter’s classification, and Moshfeghi et al. [22] similarly reported no significant differences in ramus height across Pell and Gregory classes. These findings indicate that while impaction depth may be associated with ramus morphology in some populations, the relationship is not consistently observed across classification systems.

Several methodological factors likely contributed to the observed heterogeneity. Although all included studies utilized panoramic radiography, substantial variability existed in landmark definitions, including condylion–gonion distances, sigmoid notch–antegonion measurements, and tangent‐based reference points. Differences in population characteristics (including geographic and ethnic variation), sex distributions, age ranges, imaging calibration procedures, impaction classification systems, and unilateral or bilateral impaction patterns may have further contributed to variability in effect estimates. In addition, one study was identified as potentially involving overlapping participant cohorts with a related publication, and this was addressed by excluding the duplicate dataset from the quantitative synthesis. Furthermore, studies varied in their unit of analysis, and individual participant‐level data were unavailable to permit adjustment for potential within‐subject clustering in studies that may have included bilateral mandibular third molars.

Additional sources of heterogeneity may be related to imaging‐ and measurement‐related factors, including variability in radiographic magnification inherent to panoramic imaging, differences in examiner calibration and measurement reliability across studies, and population‐specific anatomical variation. In addition, although formal assessment of publication bias was not performed due to the limited number of included studies in each meta‐analysis, the possibility of publication bias cannot be excluded.

Many studies also relied on unadjusted mean comparisons with limited control for confounding factors such as age and sex. Subgroup analyses according to study design demonstrated persistent heterogeneity within retrospective studies and did not substantially explain the variability observed in ramus height estimates, suggesting that additional methodological and population‐related factors may contribute to the observed between‐study differences.

Sensitivity analyses indicated that although the direction of effect remained consistent across leave‐one‐out analyses, the magnitude of the pooled ramus height estimate showed some variation, and statistical significance was attenuated following omission of certain influential studies. Given the limited number of studies available for quantitative synthesis and the inconsistent reporting of potentially relevant variables, further subgroup analyses or metaregression approaches were considered unlikely to yield robust estimates. Future studies employing standardized measurement protocols, more homogeneous populations, and longitudinal designs may help clarify the influence of mandibular ramus morphology on third molar eruption and impaction.

Overall, the available evidence suggests that reduced mandibular ramus height may be more consistently associated with mandibular third molar impaction than ramus width in the currently available literature, although this relationship appears to vary across populations, study designs, imaging approaches, and measurement protocols. While sexual dimorphism in ramus dimensions is well established, its role in impaction risk remains unclear. The substantial heterogeneity observed across studies, which was only partially explained by differences in study design, highlights the need for standardized measurement protocols and well‐designed longitudinal studies to better elucidate the role of mandibular ramus morphology in third molar eruption and impaction.

## 5. Conclusion

The findings of this systematic review and meta‐analysis suggest that mandibular ramus dimensions show variable associations with mandibular third molar impaction. Reduced ramus height demonstrated a more consistent relationship with impaction than ramus width, supported by both qualitative findings and pooled quantitative evidence. In contrast, ramus width exhibited highly inconsistent associations across studies and did not demonstrate a significant pooled effect. Sex‐related analyses confirmed the presence of sexual dimorphism in ramus morphology; however, its influence on impaction risk was not uniform. Evidence relating ramus dimensions to impaction classification was limited and inconsistent. Overall, these findings suggest that although reduced ramus height may be associated with mandibular third molar impaction, it should not be interpreted as an independent clinical predictor. The substantial methodological heterogeneity across studies, together with a wide prediction interval in the pooled analysis, limits definitive conclusions, underscoring the need for standardized measurement protocols and well‐designed longitudinal studies to better elucidate the role of mandibular ramus morphology in third molar impaction.

## Author Contributions

**Nazanin Salmani**: data curation, methodology, investigation, formal analysis, project administration, writing – original draft, visualization. **Hooman Shafaee**: investigation, project administration, supervision, validation. **Mostafa Abtahi**: conceptualization, supervision, validation. **Shayan Yousefi**: resources, writing – original draft. **Amirhossein Rahat Dahmardeh and Seyed MohammadMobin Mousavi**: investigation, writing – original draft. **Mahdi Naseri Takaloo and Fatemeh Elahi**: writing – review and editing.

## Funding

This research received no specific grant from any funding agency in the public, commercial, or not‐for‐profit sectors.

## Disclosure

All authors have read and approved the final version of the manuscript. Nazanin Salmani, as the corresponding author and manuscript guarantor, had full access to all of the data in this study and takes complete responsibility for the integrity of the data and the accuracy of the data analysis.

## Ethics Statement

Ethical approval and informed consent were not required for this study because it was a systematic review and meta‐analysis based exclusively on data extracted from previously published studies and did not involve direct participation of human subjects or access to identifiable patient information.

## Conflicts of Interest

The authors declare no conflicts of interest.

## Supporting Information

Additional supporting information can be found online in the Supporting Information section.

## Supporting information


**Supporting Information 1** Table S1: Characteristics of Included studies

**Supporting Information 2** Figure S1: Leave‐one‐out analysis for ramus height including nine studies, using a random‐effects model (restricted maximum likelihood estimator with Hartung–Knapp adjustment). Each panel shows the pooled effect after sequential exclusion of one study.

**Supporting Information 3** Figure S2: Leave‐one‐out analysis for ramus width including six studies, using a random‐effects model (restricted maximum likelihood estimator with Hartung–Knapp adjustment). Each panel shows the pooled effect after sequential exclusion of one study.

**Supporting Information 4** Table S2: Item‐level JBI risk‐of‐bias assessment of included studies.

## Data Availability

The authors confirm that the data supporting the findings of this study are available within the article and its Supporting Information, including Supporting Information 1: Table S1, Supporting Information 2: Figure S1, Supporting Information 3: Figure S2, and Supporting Information 4: Table S2.
